# Study on Damage Statistical Constitutive Model of Triaxial Compression of Acid-Etched Rock under Coupling Effect of Temperature and Confining Pressure

**DOI:** 10.3390/ma14237414

**Published:** 2021-12-03

**Authors:** Youliang Chen, Peng Xiao, Xi Du, Suran Wang, Zhoulin Wang, Rafig Azzam

**Affiliations:** 1 Department of Civil Engineering, School of Environment and Architecture, University of Shanghai for Science and Technology, Shanghai 200093, China; chenyouliang2001@163.com (Y.C.); xi.du@unsw.edu.au (X.D.); 2 Department of Engineering Geology and Hydrogeology, RWTH Aachen University, 52064 Aachen, Germany; azzam@lih.rwth-aachen.de; 3 School of Civil and Environmental Engineering, University of New South Wales, Sydney 2052, Australia; 4 Department of Underground Architecture and Engineering, Tongji University, Shanghai 200093, China; wangsuran@tongji.edu.cn; 5 Shanghai Key Laboratory of Engineering Structure Safety, SRIBS, Shanghai 200032, China; wangzl@usst.edu.cn

**Keywords:** rock mechanics, acid corrosion, coupling effect of temperature and confining pressure, Weibull distribution, damage variable, constitutive model

## Abstract

Based on Lemaitre’s strain equivalence hypothesis theory, it is assumed that the strength of acid-etching rock microelements under the coupling effect of temperature and confining pressure follows the Weibull distribution. Under the hypothesis that micro-element damage meets the D-P criterion and based on continuum damage mechanics and statistical theory, chemical damage variables, thermal damage variables and mechanical damage variables were introduced in the construction of damage evolution equations and constitutive models for acid-etching rocks considering the coupled effects of temperature and confining pressure. The required model parameters were obtained by theoretical derivation, and the model was verified based on the triaxial compression test data of granite. Comparing the experimental stress-strain curve with the theoretical stress-strain curve, the results show that they were in good agreement. By selecting reasonable model parameters, the damage statistical constitutive model can accurately reflect the stress-strain curve characteristics of rock in the process of triaxial compression. The comparison between the experimental and theoretical results also verifies the reasonableness and reliability of the model. This model provides a new rock damage statistical constitutive equation for the study of rock mechanics and its application in engineering, and has certain reference significance for rock underground engineering.

## 1. Introduction

With the continuous development of the global economy, the consumption of fossil fuels such as coal, oil and natural gas is increasing, and the emission of pollutants into the atmosphere is rising sharply, leading to deteriorating environmental pollution. In underground engineering, the geological conditions of rocks are very complex. In practical problems such as underground disposal of nuclear waste, underground energy storage, underground carbon dioxide storage, geothermal development, oil and natural gas exploitation, on the one hand, the rocks expand and crack when heated, on the other hand, the rocks react with aqueous chemical solutions to form holes, which greatly changes their mechanical properties, aggravate the damage evolution and seriously affect the long-term stability of the project. Therefore, it is of great practical significance to study the deterioration law and damage mechanism of mechanical properties of rock under high temperature chemical confining pressure for rock engineering construction. Meanwhile, it is of great theoretical significance to establish the damage constitutive model of rock under the coupling action of high temperature, chemistry and confining pressure. With the massive emission of carbon dioxide, acid rain has become a global environmental concern [1]. At the same time, with the continuous advancement of research in related fields of deep underground rock engineering, the influence of the chemical corrosion environment on the mechanical properties of rocks has attracted the attention of many associated scholars. Rock materials are affected in many aspects such as physics, chemistry and mechanics in an acidic environment. The corrosion phenomenon caused by the degradation of rock properties until destruction is a relatively slow chemical process, making the rock mineral composition, microstructure and mechanical properties change, resulting in undesirable engineering effects. Therefore, it has become an urgent task to understand the changes and damage evolutionary laws of rock mechanics under an acidic environment.

For a long time, the research on the effect of water-chemical solutions on rocks has attracted the attention of many scholars. It has achieved tremendous progress and fruitful results [2,3,4,5,6,7,8,9,10,11]. Some scholars have conducted experimental studies on the influence of temperature on rock physical and mechanical parameters, such as rock peak strength, Young’s modulus, Poisson’s ratio, and porosity, etc., and investigated the change of pore structure and mechanical properties of limestone exposed to acid solutions by series of triaxial and cyclic load tests [3,4,5,6,7,8,9,10,11,12,13,14,15,16,17,18,19,20]. However, only under the action of temperature and chemical solution, the effect of rock on its mechanical properties is very different from the effect of the coupling of the two. In recent years, some scholars have also carried out experimental studies on the action of chemical environment and temperature on the physical and mechanical properties of rocks [21,22,23,24,25]. For rock, the core issue of its strength theory is the constitutive model. In fact, the rock damage theory is a theory to study the damage evolution law and damage of damaged materials. The core issue is the damage model, that is, the problem of establishing damage variables and their transformation. With the development of statistical damage mechanics theory, the damage variable of the rock can be defined by the assumption that the micro-element strength follows the statistical distribution [26,27]. Based on these principles and methods, a series of damage statistical constitutive models of rock under the coupled effect of temperature and external load has been established. The corresponding chemical damage model was proposed through the quantitative description of the chemical damage mechanism of chemical solute erosion of rock [28,29]. Considering the strong nonlinearity of the peak intensity, a negative exponential empirical model was proposed to describe the peak intensity [30,31]. The effects of temperature and strain rate on peak stress, elastic modulus and energy absorption were discussed, and the damage model of rock was established to describe the thermal-mechanical coupling problem of rock accurately [32]. Using continuous damage theory and statistical methods, the modulus of elasticity was chosen as the damage variable to deduce the damage model of sandstone under the effect of temperature and hydraulic force, and a model for the damage constitution of sandstone considering the effect of temperature and hydraulic force was established [33]. An experimental study on the influence of the contact area of the undercut anchor head face on the rock physical and mechanical parameters was carried out [34].

The above research has played a positive role in analyzing the influence and damage law of rock under the effect of acidic environment and temperature. However, there are still some deficiencies, the current research direction are still mainly based on the impact of TMC on the macro-mechanical properties of rocks, in the established rock damage constitutive model, the rock micro element strength probability model and strength criterion are relatively single, and lack the constitutive properties of acid-corroded rocks. There are few studies on the damage mechanical properties of rock by establishing TMC coupled damage constitutive model, and most of them do not consider the effect of confining pressure. The study of the model requires further exploration. In view of this, the deterioration degree and damage evolution law of rock materials were described by damage variables. The damage evolution equation and damage constitutive model of rock under TMC coupling were established by using continuous damage mechanics theory and D-P criterion. Finally, the reliability of the model and the method for determining model parameters is verified by comparing with the stress-strain curve obtained by other experiments.

## 2. Establishment of Damage Constitutive Model of Rock under the Coupling Action of Acid Etching-Temperature-Confining Pressure

### 2.1. Define of Chemical Damage Variables

Yang et al. [35] defined a damage variable based on the CT (Computer Tomography) number of rocks:

(1) $$D_{\mathrm{CT}}=-\frac{1}{m_{0}^{2}}\frac{\Delta \rho }{\rho_{i}}$$

where: m_0_ is the spatial resolution of the CT machine; *ρ*_i_ is the density of the rock in the undamaged state; Δ*ρ* is the change in density during the evolution of rock damage.

CT technology is the product of the combination of radiation technology and computer technology. The basic principle is that the X-rays emitted by the CT-ray source can penetrate the material to be tested, and the penetrating ability of X-rays of different wavelengths is different, and the absorption ability of X-rays of the same wavelength is different for different materials. The quantitative description of CT is the number of CT, the inventor of the number of CT N. Hounsfield defined the CT numbers of air, water and ice as −1000, 0 and −100, respectively. Therefore, there is a certain relationship between the X-ray absorption coefficient μ value of the detected object and the CT number.

Li et al. [11] combined with Yang et al. [36] based on the damage variable of rock CT number to derive the acid-corroded sandstone damage variable *D*_C_ based on CT number:

(2) $$D_{C}=\frac{1}{2}\left(D_{C1}+D_{C2}\right)=1-\frac{E\left(\rho_{1}\right)+E\left(\rho_{2}\right)}{2\rho_{0}}$$

(3) $$E(\rho )=\frac{1000+H}{1000+H_{r}}\rho_{r}$$

Combining formula (2) and formula (3) can get the damage variable *D*_C_:

(4) $$D_{C}=1-\frac{\rho_{r}\left(2000+H_{1}+H_{2}\right)}{2\rho_{0}\left(1000+H_{r}\right)}$$

where: *ρ*_r_, *ρ*_0_, *H*_1_, *H*_2_, *H*_r_ are the density of the sandstone matrix material (g/cm^3^), the density of the undamaged sandstone (g/cm^3^), the CT numbers of the sandstone in the corroded area, the uncorroded area and the sandstone matrix material number, respectively.

### 2.2. Definition of Thermal Damage Variables

When the rock material encounters high temperatures, the generated thermal stress causes the rock and mineral particles to expand and squeeze each other, causing the cracks to expand and penetrate. Therefore, to construct a rock damage evolution equation that considers the coupling effect of temperature and confining pressure, it is necessary to introduce a and Function variables related to temperature. According to the theory of macroscopic damage mechanics, temperature damage to rock can be characterized by macroscopic mechanical parameters [37]. Through a large amount of literature and test data, it can be concluded that temperature has a softening effect on rock, that is, as temperature increases, the elastic modulus of rock decreases. Therefore, the elastic modulus can be used to define the thermal damage variable, namely

(5) $$D_{T}=1-\frac{E_{T}}{E_{0}}$$

where: *D_T_* is the rock thermal damage variable, *E_T_* is the rock elastic modulus at temperature *T* and *E*_0_ is the rock elastic modulus at room temperature.

### 2.3. Definition of Force Damage Variables

There are various definitions for damage variables. Because material damage is the cause of changes in the material’s meso-structure and certain macro-physical properties, to some extent, the destruction of rock materials is the process of damage accumulation, the benchmark for measuring damage can be selected from both the microscopic and macroscopic aspects. The damage of micro-elements in rock under load is generally random. Use the ratio of the number of damaged cells n under a certain stress level *q* to the total number of cells *N* under the initial state to define the damage variable *D*, namely

(6) $$D=\frac{n}{N}$$

According to the Krajcinovic model, the damage variable is the failure probability *P* of the element [38]. If the probability density function of the element failure is ϕ(x), then *P* is the cumulative distribution function of *F*, namely

(7) $$D_{m}=P=\int_{0}^{F}\phi (x)dx$$

Based on the strength of the rock micro-element obeys the Weibull distribution, and its probability density function is:

(8) $$\phi (F)=\frac{m}{F_{0}}{\left(\frac{F}{F_{0}}\right)}^{m-1}\exp\left[-{\left(\frac{F}{F_{0}}\right)}^{m}\right]$$

where: *F* represents the intensity of the infinitesimal element, *m* and *F*_0_ are the parameters of the Weibull distribution.

According to Equation (7), the damage variable of the rock under external load is

(9) $$D_{m}=\int_{0}^{F}\phi (x)dx=1-\exp\left[-{\left(\frac{F}{F_{0}}\right)}^{m}\right]$$

### 2.4. Definition of the Total Damage Variable of the Rock under the Coupling Action of Acid Corrosion-Temperature-Confining Pressure 

Assuming that the effective volumes of undamaged rock, temperature damaged rock and temperature-confining pressure coupling effect damage rock are *V*_0_, *V*_1_ and *V*_2_, respectively, then

(10) $$D_{T}=1-\frac{V_{1}}{V_{0}}$$

(11) $$D_{m}=1-\frac{V_{2}}{V_{1}}$$

From Equations (10) and (11), namely

(12) $$D_{S}=D_{m}+D_{T}-D_{m}D_{T}$$

where: *D_S_* is the total damage variable of the rock under the coupling effect of temperature and confining pressure.

In summary, the total damage variable of rock under the coupling action of acid corrosion-temperature-confining pressure can be obtained:

(13) $$D=D_{C}+D_{S}-D_{C}D_{S}=D_{C}+D_{T}+D_{m}-D_{T}D_{m}-D_{C}D_{T}-D_{C}D_{m}+D_{C}D_{T}D_{m}$$

Substituting formula (4), (5) and formula (9) into formula (13) can obtain the total damage variable of rock under the coupling action of acid corro-sion-temperature-confining pressure:

(14) $$D=1-\frac{\rho_{r}\left(2000+H_{1}+H_{2}\right)}{2\rho_{0}\left(1000+H_{r}\right)}\frac{E_{T}}{E_{0}}\exp\left[-{\left(\frac{F}{F_{0}}\right)}^{m}\right]$$

### 2.5. Definition of Rock Micro-Element Strength under Confining Pressure

The failure of rock under external load is generally shear failure, namely

(15) $$f(\sigma^{*})=k_{0}$$

where: σ^*^ is the effective stress of the rock and *k*_0_ is a constant.

Based on the strength of the micro-element of the rock obeys the DP criterion, namely

(16) $$F=f(\sigma^{*})=\alpha I_{1}+\sqrt{J_{2}}$$

where: *α* is the strength parameter of the rock element, *I*_1_ and *J*_2_ are the first invariant of stress and the second invariant of deviator stress, and *α*, *I*_1_ and *J*_2_ are

(17) $$\alpha =\frac{\sin\varphi }{\sqrt{9+3\sin^{2}\varphi }}$$

(18) $$I_{1}=\sigma_{1}^{*}+\sigma_{2}^{*}+\sigma_{3}^{*}$$

(19) $$J_{2}=\frac{{\left(\sigma_{1}^{*}-\sigma_{2}^{*}\right)}^{2}+{\left(\sigma_{2}^{*}-\sigma_{3}^{*}\right)}^{2}+{\left(\sigma_{3}^{*}-\sigma_{1}^{*}\right)}^{2}}{6}$$

respectively. *φ* is the friction angle of the rock.

(20) $$\left.\begin{array}{l} \varepsilon_{1}=(\sigma_{1}^{*}-2v\sigma_{3}^{*})/E\\ \sigma_{1}^{*}=\frac{\sigma_{1}}{1-D_{m}}\\ \sigma_{2}^{*}=\frac{\sigma_{2}}{1-D_{m}}\\ \sigma_{3}^{*}=\frac{\sigma_{3}}{1-D_{m}}\\ 1-D_{m}=\frac{\left(\sigma_{1}-2v\sigma_{3}\right)}{E\varepsilon_{1}} \end{array}\right\}$$

Replace *I*_1_ and *J*_2_ with nominal stress *σ_i_* representation, namely

(21) $$I_{1}=E\varepsilon_{1}(\sigma_{1}+2\sigma_{3})/(\sigma_{1}-2v\sigma_{3})$$

(22) $$\sqrt{J_{2}}=E\varepsilon_{1}(\sigma_{1}-\sigma_{3})/\sqrt{3}(\sigma_{1}-2v\sigma_{3})$$

From formulas (16), (17), (21), (22), namely

(23) $$F=f(\sigma^{*})=\alpha I_{1}+\sqrt{J_{2}}=\frac{E\varepsilon_{1}\left[\sin\varphi \left(\sigma_{1}+2\sigma_{3}\right)+\sqrt{3+\sin^{2}\varphi }(\sigma_{1}-\sigma_{3})\right]}{\sqrt{9+3\sin^{2}\varphi }(\sigma_{1}-2v\sigma_{3})}$$

### 2.6. Damage Evolution Equation of Rock under the Coupling Action of Acid Etching-Temperature-Confining Pressure

Substituting Equation (23) into Equation (14), namely

(24) $$D=1-\frac{\rho_{r}\left(2000+H_{1}+H_{2}\right)}{2\rho_{0}\left(1000+H_{r}\right)}\frac{E_{T}}{E_{0}}\exp\left\{-{\left\{\frac{E_{T}\varepsilon_{1}\left[\sin\varphi \left(\sigma_{1}+2\sigma_{3}\right)+\sqrt{3+\sin^{2}\varphi }(\sigma_{1}-\sigma_{3})\right]}{F_{0}\sqrt{9+3\sin^{2}\varphi }(\sigma_{1}-2v\sigma_{3})}\right\}}^{m}\right\}$$

### 2.7. Damage Constitutive Model of Rock under the Coupling Action of Acid Etching-Temperature-Confining Pressure

Based on the Lemaitre strain equivalence principle and the concept of effective stress, the rock damage constitutive relationship can be established as follows:

(25) $$\sigma_{i}^{*}=\frac{\sigma_{i}}{1-D};i=1,2,3$$

where: $\sigma_{i}^{*}$ is the effective stress of the rock, *σ_i_* is the nominal stress of the rock, and *D* is the total damage variable of the rock. According to the generalized Hooke’s law

(26) $$\varepsilon_{i}^{*}=\left[\sigma_{i}^{*}-v(\sigma_{j}^{*}+\sigma_{k}^{*})\right]/E_{T}$$

where: (*i,j,k*) is (1,2,3), *E_T_*, *v*, $\varepsilon_{i}^{*}$ are the rock elastic modulus at temperature *T*, Poisson’s ratio, the effective strain corresponding to the effective stress $\sigma_{i}^{*}$, respectively.

Based on the deformation coordination conditions:

(27) $$\varepsilon_{i}^{*}=\varepsilon_{i}^{}$$

From Equations (25)–(27), we can get:

(28) $$\sigma_{1}=E_{T}\varepsilon_{1}(1-D)+v(\sigma_{2}+\sigma_{3})$$

The initial axial strain generated under the initial confining pressure is

(29) $$\sigma_{1t}=\sigma_{1}-\sigma_{3}$$

(30) $$\varepsilon_{10}=\frac{1-2v}{E_{T}}\sigma_{3}$$

(31) $$\varepsilon_{1}=\varepsilon_{1t}+\varepsilon_{10}$$

where: *ε*_1_, *ε*_1t_, *ε*_10_ are the actual axial strain, the measured axial strain, the initial axial strain, respectively, σ_1t_ is the axial deflection stress.

Substituting Equations (24) and (29)–(31) into Equation (28), the statistical constitutive model of triaxial compression of rock under the coupling action of acid etching-temperature-confining pressure can be obtained:

(32) $$\begin{array}{l} \sigma_{1t}=\left[E_{T}\varepsilon_{1t}+(1-2v)\sigma_{3}\right]\frac{\rho_{r}\left(2000+H_{1}+H_{2}\right)}{2\rho_{0}\left(1000+H_{r}\right)}\frac{E_{T}}{E_{0}}\exp\left\{-{\left\{\frac{\left[E_{T}\varepsilon_{1t}+(1-2v)\sigma_{3}\right]\left[(\sigma_{1t}+3\sigma_{3})\sin\varphi +\sigma_{1t}\sqrt{3+\sin^{2}\varphi }\right]}{F_{0}\sqrt{9+3\sin^{2}\varphi }\left[\sigma_{1t}+(1-2v)\sigma_{3}\right]}\right\}}^{m}\right\}\\ +(2v-1)\sigma_{3} \end{array}$$

## 3. Determination of Model Parameters

The model parameter *ρ*_r_, *ρ*_0_, *H*_1_, *H*_2_, *H*_r_ is obtained through the rock test and rock CT scan test. The expression of *E_T_* can be expressed as:

*E_T_* = a*T*^2^ + b*T* + c (33)

where: a, b, and c are constants obtained by fitting experimental data.

The peak stress *σ*_p_ and the corresponding peak strain *ε*_p_ of the rock meet the following two geometric conditions:

(34) $$\varepsilon =\varepsilon_{p},\sigma_{1}=\sigma_{p}$$

(35) $$\varepsilon =\varepsilon_{p},\frac{d\sigma_{1}}{d\varepsilon_{1}}=0$$

First, from Equations (32) and (34), we can get:

(36) $$\frac{\rho_{r}\left(2000+H_{1}+H_{2}\right)}{2\rho_{0}\left(1000+H_{r}\right)}\frac{E_{T}}{E_{0}}\exp\left[-{\left(\frac{F_{\mathrm{sc}}}{F_{0}}\right)}^{m}\right]=\frac{\sigma_{p}+(1-2v)\sigma_{3}}{E_{T}\varepsilon_{p}+(1-2v)\sigma_{3}}$$

among them,

(37) $$F=\frac{\left[E_{T}\varepsilon_{1t}+(1-2v)\sigma_{3}\right]\left[(\sigma_{1t}+3\sigma_{3})\sin\varphi +\sigma_{1t}\sqrt{3+\sin^{2}\varphi }\right]}{\sqrt{9+3\sin^{2}\varphi }\left[\sigma_{1t}+(1-2v)\sigma_{3}\right]}$$

Secondly, the derivative of Equation (32) and combine Equation (35).

Take the partial derivative of Equation (32) to get

(38) $${\left.\frac{\partial \sigma_{1t}}{\partial \varepsilon_{1t}}\right|}_{\begin{array}{l} \sigma_{1t}=\sigma_{p}\\ \varepsilon_{1t}=\varepsilon_{p} \end{array}}=\frac{\rho_{r}\left(2000+H_{1}+H_{2}\right)}{2\rho_{0}\left(1000+H_{r}\right)}\frac{E_{T}}{E_{0}}\exp\left[-{\left(\frac{F}{F_{0}}\right)}^{m}\right]\left\{E_{T}-\frac{m\left[E_{T}\varepsilon_{p}+(1-2v)\sigma_{3}\right]}{F_{\mathrm{sc}}}{\left(\frac{F_{\mathrm{sc}}}{F_{0}}\right)}^{m}\frac{\partial F_{\mathrm{sc}}}{\partial \varepsilon_{1t}}\right\}$$

Know from formula (35)

(39) $$E_{T}-\frac{m\left[E_{T}\varepsilon_{p}+(1-2v)\sigma_{3}\right]}{F_{\mathrm{sc}}}{\left(\frac{F_{\mathrm{sc}}}{F_{0}}\right)}^{m}\frac{\partial F_{\mathrm{sc}}}{\partial \varepsilon_{1t}}=0$$

From Equation (37) can be obtained:

(40) $${\left.\frac{\partial F_{\mathrm{sc}}}{\partial \varepsilon_{1t}}\right|}_{\begin{array}{l} \sigma_{1t}=\sigma_{p}\\ \varepsilon_{1t}=\varepsilon_{p} \end{array}}=\frac{E_{T}\left[(\sigma_{p}+3\sigma_{3})\sin\varphi +\sigma_{p}\sqrt{3+\sin^{2}\varphi }\right]}{\sqrt{9+3\sin^{2}\varphi }\left[\sigma_{p}+(1-2v)\sigma_{3}\right]}$$

From Equations (39) and (40), we can get

(41) $${\left(\frac{F_{\mathrm{sc}}}{F_{0}}\right)}^{m}=\frac{F_{\mathrm{sc}}\sqrt{9+3\sin^{2}\varphi }\left[\sigma_{p}+(1-2v)\sigma_{3}\right]}{m\left[E_{T}\varepsilon_{p}+(1-2v)\sigma_{3}\right]\left[(\sigma_{p}+3\sigma_{3})\sin\varphi +\sigma_{p}\sqrt{3+\sin^{2}\varphi }\right]}=\frac{1}{m}$$

From formula (36) and (41), the parameters *m* and *F*_0_ can be obtained:

(42) $$m=\frac{1}{\ln\frac{E_{T}}{E_{0}}\frac{\rho_{r}\left(2000+H_{1}+H_{2}\right)}{2\rho_{0}\left(1000+H_{r}\right)}\left[\frac{E_{T}\varepsilon_{p}+(1-2v)\sigma_{3}}{\sigma_{p}+(1-2v)\sigma_{3}}\right]}$$

(43) $$F_{0}=\frac{\left[E_{T}\varepsilon_{p}+(1-2v)\sigma_{3}\right]\left[(\sigma_{p}+3\sigma_{3})\sin\varphi +\sigma_{p}\sqrt{3+\sin^{2}\varphi }\right]}{\sqrt{9+3\sin^{2}\varphi }\left[\sigma_{p}+(1-2v)\sigma_{3}\right]}m^{\frac{1}{m}}$$

Substitute the model parameters determined above into Equation (32), the damage constitutive model of rock under the coupling action of acid etching-temperature-confining pressure can be obtained.

## 4. Verification of Damage Statistical Constitutive Model of Rock under Triaxial Compression

This paper used the research results of Min et al. [39] on uniaxial and triaxial compression tests of granite after high temperature to verify the accuracy and applicability of the model. The specific method is as follows:

Min et al. [39]; in the study of the triaxial compression test and the evolution of mechanical properties of Beishan granite, the MXQ1700 box-type atmosphere furnace was used to treat the rock samples at a high temperature with a heating rate of 5 °C/min and a target temperature of 25 °C, 200 °C, 400 °C, 600 °C, 800 °C, after the temperature of the rock sample reaches the target temperature, keep it at a constant temperature for 2 h. After the program is stopped, turn off the power and cool naturally in the high-temperature furnace. The confining pressures of the rock triaxial compression test are 1 MPa, 5 MPa, 10 MPa, 15 MPa and 25 MPa, respectively. The original physical and mechanical parameters of Beishan granite under triaxial compression are shown in Table 1. The test curve was obtained by performing triaxial compression tests at different temperatures and confining pressures [39] (temperatures are 25 °C, 200 °C, 400 °C, 600 °C, 800 °C, respectively, and confining pressures are 1 MPa, 5 MPa, 10 MPa, 15 MPa, 25 MPa, respectively).

Substitute the parameters in Table 1 into Equations (30) and (31), the parameters *m* and *F*_0_ are shown in Table 2.

### Comparison and Analysis of Results

**Figure 1:**
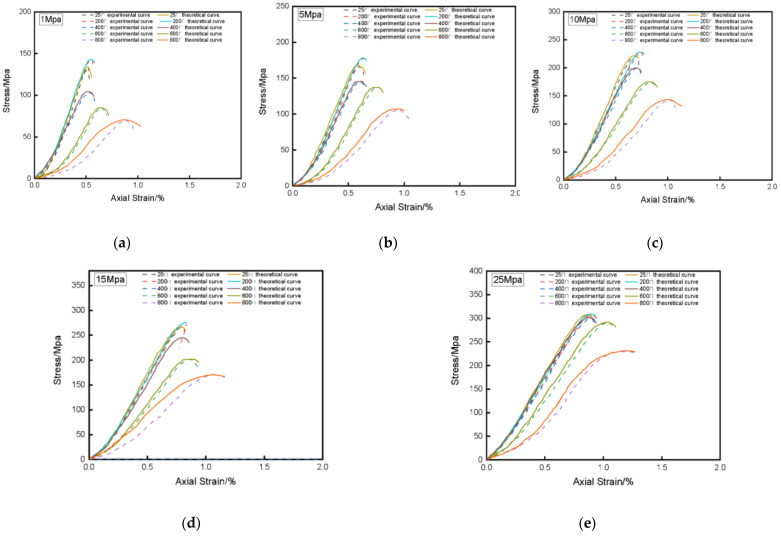
Experimental and theoretical stress-strain curves under different confining pressures. (**a**) 1 Mpa; (**b**) 5 Mpa; (**c**) 10 Mpa; (**d**) 15 Mpa; (**e**) 25 Mpa.

**Figure 2:**
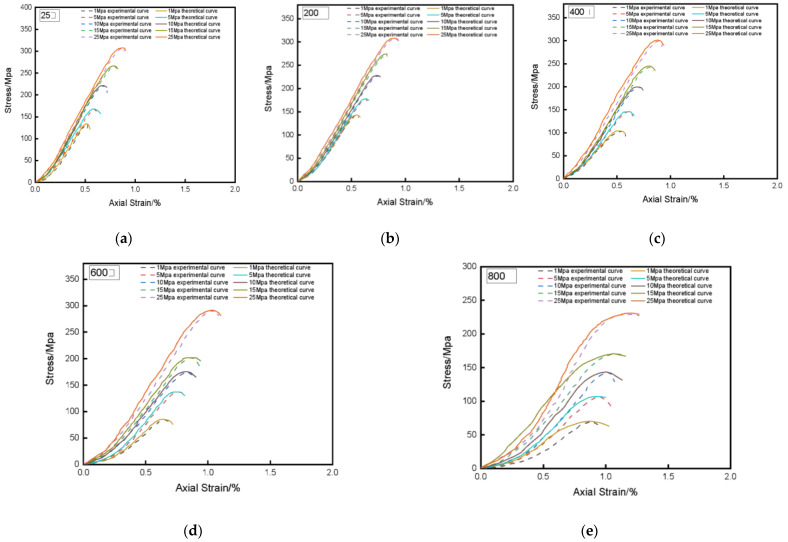
Experimental and theoretical stress-strain curves at different temperatures. (**a**) 25 °C; (**b**) 200 °C; (**c**) 400 °C; (**d**) 600 °C; (**e**) 800 °C.

Compare the peak values of stress-strain test curves and theoretical curves of granite under different temperatures and confining pressures, as shown in Table 3. In order to display the comparison results more intuitively, compare the experimental stress-strain curve with the theoretical stress-strain curve under different confining pressures and different temperatures, as shown in Figure 1 and Figure 2. By comparing the experimental stress-strain curve with the theoretical stress-strain curve, indicate that the established damage statistical constitutive model can better reflect the stress-strain characteristics of the rock during triaxial compression. Under the same confining pressure, the peak strain at the peak point of the theoretical curve of the rock damage constitutive model established in this paper does not change much with temperature, which is consistent with the actual situation. The peak stress will gradually decrease with the increase of temperature. It can be seen from Figure 1 that under the same confining pressure, the peak stress of granite gradually decreases with increasing temperature. It can be seen from Figure 2 that under the same temperature, the peak stress of granite increases with increasing confining pressure, which is consistent with the experimental results. The theoretical curve can reflect the rock the trend of strength and deformation with temperature and confining pressure, which further illustrates the applicability of the constitutive model applicability and accuracy. 

## 5. Conclusions

In this paper, by introducing different damage variables and selecting the D-P criterion, a damage constitutive model including the damage evolution equation under the action of acid etching, temperature and confining pressure is established. The acid corrosion-high temperature-confining pressure of rock constitutive model established can well predict the stress-strain characteristics of the rock, the peak point of the model is consistent with the test peak point, and the theoretical curve can reflect the variation trend of rock strength and deformation with temperature and confining pressure. At the same time, it also shows that the damage model, including the rock damage evolution equation established in this paper, can fully reflect the characteristics that rock strength depends on confining pressure. The main conclusions are as follows: 

(1) The theoretical stress-strain curve of the established model is compared with the stress-strain curve obtained from the experiment. Indicates that the established constitutive model is more accurate. The rock damage constitutive model established in this paper can better reflect the stress-strain characteristics of rock during triaxial compression, and has certain practical significance for mining and nuclear waste storage in underground high-temperature chemical environments. It is of great significance in the actual work of predicting the damage of geotechnical engineering materials.

(2) The peak strain of the theoretical stress-strain curve of the model does not change much with the change of temperature, while the peak stress will fluctuate slightly with the change of temperature. The peak point of the theoretical stress-strain curve and the peak point of the experimental stress-strain curve are in good agreement with the change of temperature.

(3) The model parameters *F*_0_ are affected by the confining pressure and peak stress of the rock, and have certain mechanical significance. It further explains the correctness and rationality of the assumptions in the process of model building.

(4) The rock constitutive model established in this paper has the following advantages: there is no specific rock type parameter, and the required model parameters can be obtained through conventional triaxial tests; it has nothing to do with the rock type, is a convenient engineering application, and has wide applicability.

## Figures and Tables

**Table 1:** Original physical and mechanical parameters of granite under triaxial compression.

*T*/°C	*σ*_3_/MPa	*E_T_*/GPa	*v*	*φ*/°
25	1	36.43	0.191	49.68
	5	37.30	0.199	
	10	38.70	0.220	
	15	41.10	0.234	
	25	41.80	0.245	
200	1	35.55	0.212	48.96
	5	38.14	0.243	
	10	39.30	0.268	
	15	42.12	0.283	
	25	42.83	0.297	
400	1	30.40	0.240	51.89
	5	33.60	0.274	
	10	37.36	0.311	
	15	39.05	0.327	
	25	40.46	0.343	
600	1	21.09	0.255	51.48
	5	26.94	0.282	
	10	28.52	0.309	
	15	31.36	0.336	
	25	34.13	0.352	
800	1	13.57	0.264	46.83
	5	17.79	0.302	
	10	21.43	0.332	
	15	23.51	0.340	
	25	27.73	0.376	

**Table 2:** Model calculation parameters of triaxial compression of granite.

*T*/°C	*σ*_3_/MPa	*σ*_p_/MPa	*ε*_p_/%	*m*	*F*_0_/MPa
25	1	134.02	0.520	2.901	222.591
	5	168.50	0.611	3.362	270.240
	10	221.16	0.694	5.266	308.440
	15	267.10	0.805	4.797	387.323
	25	308.19	0.887	5.614	433.885
200	1	143.00	0.560	3.033	232.974
	5	178.32	0.640	3.224	289.190
	10	230.00	0.745	4.217	343.619
	15	274.40	0.819	4.462	405.912
	25	310.91	0.908	4.599	464.908
400	1	104.00	0.550	2.115	195.730
	5	147.00	0.670	2.376	270.941
	10	205.00	0.716	3.819	322.005
	15	245.00	0.821	3.787	389.997
	25	305.00	0.909	5.468	437.147
600	1	82.14	0.647	1.979	158.301
	5	135.00	0.775	2.323	251.071
	10	170.00	0.840	2.971	294.258
	15	200.00	0.881	3.160	343.006
	25	287.78	1.050	4.664	434.994
800	1	68.05	0.883	1.777	133.929
	5	104.00	0.958	2.055	200.243
	10	140.00	1.030	2.239	266.396
	15	166.83	1.110	2.287	319.621
	25	225.00	1.228	2.468	426.229

**Table 3:** Peak values of stress-strain test curves and theoretical curves of granite under different temperatures and confining pressures.

*T*/°C	*σ*_3_/MPa	*σ*_p_/MPa	*ε*_p_/%	$\sigma_{p}^{\prime }/\mathrm{MPa}$	$\varepsilon_{p}^{\prime }/\%$	R
25	1	134.02	0.520	134.00	0.528	0.998
	5	168.50	0.611	168.50	0.616	
	10	221.16	0.694	221.00	0.704	
	15	267.10	0.805	267.10	0.802	
	25	308.19	0.887	308.20	0.905	
200	1	143.00	0.560	142.90	0.572	0.999
	5	178.32	0.640	178.30	0.641	
	10	230.00	0.745	229.90	0.752	
	15	274.40	0.819	274.30	0.818	
	25	310.91	0.908	310.80	0.916	
400	1	104.00	0.550	104.00	0.549	0.998
	5	147.00	0.670	147.00	0.669	
	10	205.00	0.716	205.00	0.723	
	15	245.00	0.821	245.00	0.828	
	25	305.00	0.909	305.00	0.912	
600	1	82.14	0.647	82.15	0.645	0.999
	5	135.00	0.775	135.00	0.778	
	10	170.00	0.840	170.10	0.841	
	15	200.00	0.881	200.00	0.885	
	25	287.78	1.050	287.90	1.050	
800	1	68.05	0.883	68.06	0.891	0.997
	5	104.00	0.958	104.00	0.943	
	10	140.00	1.030	140.00	1.030	
	15	166.83	1.110	166.80	1.098	
	25	225.00	1.228	225.00	1.232	

## Data Availability

Data sharing is not applicable to this article.
